# COVID-19 Symptoms and Diagnoses among a Sociodemographically Diverse Cohort of Children from New York City: Lessons from the First Wave, Spring 2020

**DOI:** 10.3390/ijerph182211886

**Published:** 2021-11-12

**Authors:** Linda G. Kahn, Akhgar Ghassabian, Melanie H. Jacobson, Keunhyung Yu, Leonardo Trasande

**Affiliations:** 1 Departments of Pediatrics and Population Health, New York University Grossman School of Medicine, New York, NY 10016, USA; 2 Departments of Pediatrics, Population Health, and Environmental Medicine, New York University Grossman School of Medicine, New York, NY 10016, USA; akhgar.ghassabian@nyulangone.org (A.G.); leonardo.trasande@nyulangone.org (L.T.); 3 Department of Pediatrics, New York University Grossman School of Medicine, New York, NY 10016, USA; melanie.jacobson2@nyulangone.org (M.H.J.); keunhyung.yu@nyulangone.org (K.Y.); 4 Wagner School of Public Service, New York University, New York, NY 10012, USA; 5 College of Global Public Health, New York University, New York, NY 10003, USA

**Keywords:** COVID-19, pediatrics, epidemiology, cohort study

## Abstract

Early in the pandemic, in the North American epicenter, we investigated associations between sociodemographic factors and rates of pediatric COVID-19 diagnoses in a non-clinical setting and whether symptoms varied by child age. From 20 April–31 August 2020, COVID-19-related data were collected on 2694 children aged ≤ 18 years living in households participating in the New York University Children’s Health and Environment Study. We examined differences in rates of subjective and objective diagnoses according to sociodemographic characteristics and differences in reported symptoms by child age. Children of women who were non-Hispanic White, had private health insurance, higher income, or more education were more likely to be diagnosed via WHO criteria or healthcare provider. Children of women who were Hispanic or Asian, reported low income, had less education, or were/lived with an essential worker were more likely to test positive. Older children were less likely to experience cough or runny nose and more likely to experience muscle/body aches, sore throat, headache, and loss of smell or taste than younger children. In conclusion, relying on subjective disease ascertainment methods, especially in the early stage of an outbreak when testing is not universally available, may misrepresent the true prevalence of disease among sociodemographic subgroups. Variations in symptoms by child age should be considered when determining diagnostic criteria.

## 1. Introduction

Racial/ethnic and economic disparities in COVID-19 diagnosis have been amply described among the United States (US) population [1,2,3], but datasets that inform these studies generally lack information on other sociodemographic factors. Studies of pediatric COVID-19 cases have been fewer and similarly limited, although one study has reported differences in test positivity rates according to both race/ethnicity and socioeconomic status [4]. Children’s age may also play a factor in pediatric COVID-19 susceptibility, with a US Centers for Disease Control and Prevention Morbidity and Mortality Weekly Report showing consistently higher diagnosis and reverse transcriptase polymerase chain reaction (RT-PCR) test positivity rates among older children (age 11–17 years) compared with younger ones (age 0–10 years) between March and December, 2020, based on reported cases and electronic laboratory tests [5]. Although it has been noted that children are more likely to present with mild or asymptomatic cases compared with adults [6], little is known about how the constellation of symptoms they do incur differs by age. What is known has primarily come from case reports [7] and hospitalized patients [8], not the general population.

In New York City (NYC), the earliest North American epicenter of the COVID-19 pandemic, near the peak of the initial outbreak, we surveyed a diverse, well-characterized cohort of mothers who were already enrolled in an ongoing child health study about the impact of COVID-19 on themselves and children in their households. Our objectives were to assess whether and how racial/ethnic and socioeconomic disparities were reflected in children’s case diagnoses and test positivity rates, and to examine differences in reported symptoms by child life stage between birth and 18 years.

## 2. Materials and Methods

### 2.1. Study Population

The New York University Children’s Health and Environment Study (NYU CHES) is a longitudinal pregnancy and child health study that has recruited women <18 weeks of gestation since March, 2016 who were planning to deliver at one of three NYU-affiliated hospitals: NYU Langone Hospital-Manhattan, a major acute care center for the NYC metropolitan region; Bellevue Hospital, the flagship of the largest municipal hospital system in North America; and NYU Langone Hospital-Brooklyn’s Family Health Center, the second largest federally qualified health center in the nation. Apart from being more likely to be married and of Hispanic ethnicity and less likely to be non-Hispanic Black or non-Hispanic Asian, NYU CHES participants are sociodemographically comparable to all women who give birth in NYC [9].

### 2.2. Variables

Beginning on April 20, 2020, a COVID-19 questionnaire was sent to NYU CHES participants who were currently pregnant or had delivered a live birth (*n* = 2603). Women were asked about COVID-like symptoms, COVID-19 diagnoses, and testing histories of all children aged ≤18 years in their households (not only those enrolled in our birth cohort) since March 1, the day following the first confirmed COVID-19 diagnosis in NYC [10]. Data were merged with sociodemographic information collected in prior questionnaires completed by women during their index pregnancy and from medical records.

### 2.3. Statistical Analysis

Because testing was restricted during the period when most data were collected, we calculated the cumulative prevalence of COVID-19 among children according to three metrics commonly in use at the time: (1) World Health Organization (WHO) “suspect case” criteria (acute onset of fever and cough or acute onset of any three or more of the following signs or symptoms: fever, cough, general weakness/fatigue, headache, myalgia, sore throat, coryza, dyspnoea, anorexia/nausea/vomiting, diarrhea, and altered mental status, AND residing or working in an area with high risk of transmission of virus [11]), based on reported symptoms and assuming all participants to be in a high-risk area, as New York was the epicenter of the outbreak in North America when the questionnaire was circulated; (2) reported diagnosis by a healthcare provider (“Has a healthcare provider ever told you that the child had, or might have had, COVID-19?”); and (3) reported positive test result (either via RT-PCR, as antigen tests were not yet available, or via serology: “Has the child had the nose swab test for COVID-19?” “Has the child had a blood test to see whether they already had COVID-19?”). Because of low numbers, we combined the two types of testing into a single group, although they differ in sensitivity and specificity, and results of antibody testing are dependent upon time since infection, which we were unable to ascertain. Prevalence of symptoms among cases was stratified by child age categories. In chi-square analyses (using Fisher’s exact test when warranted), we examined differences in the prevalence of children’s case diagnosis and test positivity according to women’s race/ethnicity, insurance type (public versus private), annual household income, education, and whether the woman, her partner, or another adult on whom she depended for income had a job designated as an essential service or high-risk for COVID-19. T-tests were used to examine differences in reported symptoms according to child life stage: infancy (<1 year), preschool (1–3 years, reference), elementary school (4–9 years), middle/high school (10–18 years). A sensitivity analysis was run excluding both women who responded after June 1, the end of the first wave in NYC [10] and the date when the state implemented universal testing [12], and women who reported living outside the NYC metropolitan area, who may not have been living in a high-risk area. Statistical significance was set at a two-tailed *p*-value of <0.05. All analyses were conducted using SAS 9.4 (Cary, NC, USA).

## 3. Results

**Figure 1:**
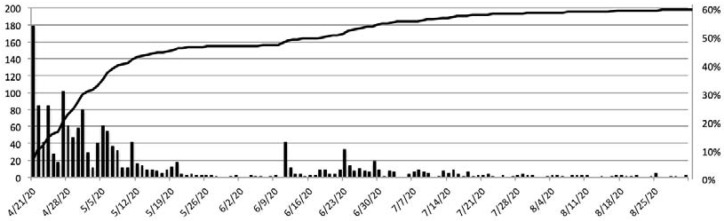
Number of responses per day and total response, NYU CHES COVID-19 questionnaire.

Sixty percent of NYU CHES participants who were sent the COVID-19 questionnaire (*n* = 1560) completed it by 31 August 2020; of those, 78.3% (*n* = 1221) responded prior to June 1, during the first wave of the epidemic in NYC and when testing was by prescription only (Figure 1).

**Figure 2:**
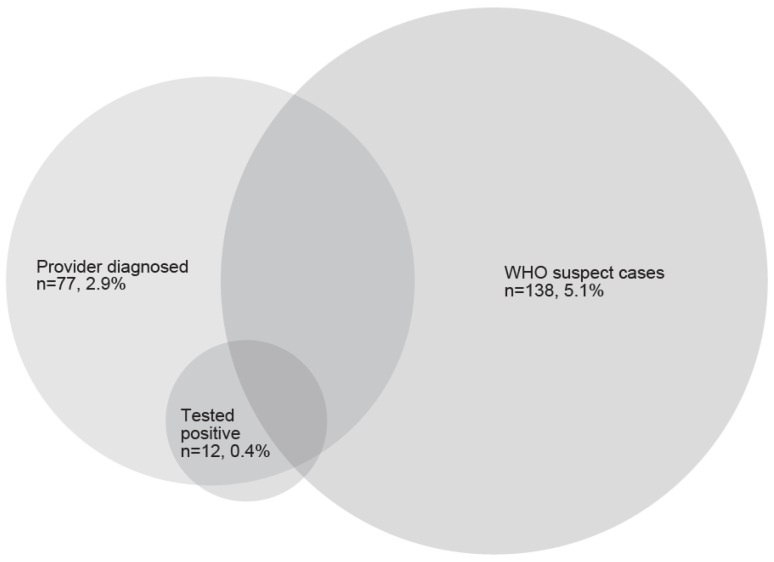
Children’s COVID-19 case diagnosis by three methods in NYU CHES, 20 April–31 August 2020 (*n* = 185).

Respondents provided data on 2694 children, of whom 185 (6.9%) met at least one case definition: 138 (5.1%) met WHO suspect case criteria, 77 (2.9%) were told by a healthcare provider that they had or might have had COVID-19, and 12 (0.4%) tested positive, either by RT-PCR or serology. There was overlap among the three groups: for example, among the 138 children who met WHO suspect case criteria based on reported symptoms, 30 (21.7%) were also diagnosed by a healthcare provider; among the 77 children diagnosed by a healthcare provider, 11 (14.3%) tested positive; and four children met all three case definitions (Figure 2).

Participants who completed the survey were comparable to the underlying cohort in terms of age, race/ethnicity, marital status, education, income, employment, and insurance status (Table S1).

Table 1 shows that the prevalence of diagnosis via WHO suspect case criteria or healthcare provider was higher among children of women who self-identified as non-Hispanic White, had private health insurance, had higher income, or were more educated than among other children. In contrast, children who lived with an adult who was an essential worker or who had a job that put them at high risk of COVID-19 were more likely than other children to test positive. Children of women who self-identified as Hispanic or non-Hispanic Asian, who reported annual household income <$30,000, or who had less than a postgraduate degree were also more likely to test positive, although differences were not statistically significant, probably because of the low prevalence of positive test results among the cohort (0.4%). The pattern for the positive test rate (# tested positive/# tested) was similar, but also not statistically significant because of small numbers. For example, the positive test rate was 20.8% for children of Hispanic women (10 positive results out of 48 tests) and 16.7% for children of non-Hispanic Asian women (1 positive result out of 6 tests) versus 5.6% for children of non-Hispanic White women (1 positive result out of 18 tests). No non-Hispanic Black children or children of other or multiple races tested positive within this time period. Younger children (≤3 years) were more likely to receive a positive provider diagnosis compared with older children. The percent of all children who tested positive and the test positivity rates were highest among children age <1 and 10–18 years although, once again, these results were not statistically significant.

A sensitivity analysis among children of participants living in the NYC metropolitan area who responded before 1 June (*n* = 1889) yielded similar results (Table S2).

**Figure 3:**
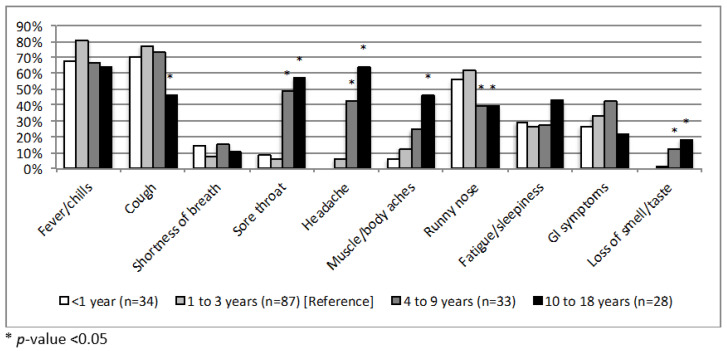
Reported symptoms among children with suspected or diagnosed COVID-19 in NYU CHES, 20 April–31 August 2020 (*n* = 182).

Among children with suspected or diagnosed COVID-19 (*n* = 185), the most prevalent symptoms were fever or chills (>60% of children from all age groups) and cough, while the least prevalent symptoms were shortness of breath and loss of smell or taste (<20% of children for each). Children age 10–18 years were less likely to experience cough than 1–3 year olds (46% versus 77%) and more likely to experience muscle/body aches (46% versus 13%). Although not statistically significant, possibly due to small sample size, a higher percentage of children age 10–18 years also experienced fatigue or sleepiness compared to younger children. School-age children (4–18 years) were more likely to experience sore throat, headache, and loss of smell or taste than 1–3 year olds, and less likely to experience runny nose (Figure 3). Fourteen of the children who met one of the three case definitions in our cohort (7.6%) were asymptomatic: none of those classified as WHO suspect cases, 13 (16.9%) of those diagnosed by a healthcare provider, and 3 (25.0%) of those who tested positive (data not shown).

Child age-specific symptom patterns were similar but not identical across the three diagnosis methods (Supplementary Materials, Figure S1a–c). For example, a higher percentage of children diagnosed via the WHO suspect case definition were reported to have had fever or chills (86%) compared with those who were diagnosed by a healthcare provider or who tested positive (55% and 67%, respectively), as fever was one of the primary WHO diagnostic criteria. Because of small numbers, we were unable to perform statistical analyses comparing age groups within each diagnostic category. As might be expected, children without a COVID-19 diagnosis had markedly lower frequencies of all symptoms (Supplementary Materials, Figure S1d,e).

## 4. Discussion

In this analysis of COVID-19 diagnoses and symptoms among NYC children at the peak of the first wave of the pandemic, we found that children who lived with non-Hispanic White women and women with higher socioeconomic status were more likely to be classified as COVID-19 cases by subjective measures (meeting WHO suspected case criteria or being diagnosed by a healthcare provider). Because there was a low prevalence of testing intrinsic to the study period and thus a low prevalence of cases ascertained by positive test result (RT-PCR or antibody: 0.4%), we were underpowered to detect statistically significant results among this subset. Nevertheless, we observed a trend toward increased diagnosis by such objective measures among children from less privileged circumstances. In particular, children in households where an adult self-identified as an essential worker or had a job that was high-risk for COVID-19 exposure were more likely to be infected when tested. Although not statistically significant, higher positive test rates were also observed among children of women who reported Hispanic ethnicity, low income, and low education. These latter findings are in keeping with the only other pediatric COVID-19 study that has addressed both race/ethnicity and socioeconomic status. In that analysis, 1000 children tested at a drive-through/walk-up testing site in Washington, DC in March–April 2020, Hispanic and non-Hispanic Black children were more likely to test positive compared with non-Hispanic White children and the test positivity rate was inversely associated with median family income [4].

Although our cohort is not a representative sample of all NYC households, test results among children living in our participants’ households accurately reflect the situation on the ground at the time of the survey. Cumulative prevalence of COVID-19 based on testing among children age ≤18 years in our study was comparable to simultaneous prevalence data available from the NYC Department of Health and Mental Hygiene (0.4% as of August 27) [13]. As expected, seeing as New York was harder hit than other parts of the country at that time, the positive test rate among our cohort was higher than among children nationwide during approximately the same period [5].

One of the strengths of our study, that it was conducted early in the pandemic, may also be the source of its superficially contradictory results, in which different subpopulations were more likely to be diagnosed via different case identification methods. During the time when most participants completed the survey, COVID-19 testing was scarce and available only by prescription. Diagnoses and referrals for testing were commonly made based on symptoms and circumstances, such as travel history or likely exposure. Those who most frequently noted COVID-19 symptoms among children in their households and who most frequently received provider diagnoses tended to be from more privileged groups. This may be because these women were more likely to seek and/or receive care, because they were more likely to report their children’s symptoms when they did receive care, or because they were more likely to have recently traveled and therefore their children were presumed to be exposed. While all of the participants in our study were insured and technically had access to pediatric care through personal physicians or hospital clinics, non-Hispanic White women and women of higher socioeconomic status may have felt more empowered to take advantage of that access. At the same time, women from less privileged groups may have faced barriers to seeking or receiving care other than insurance because of entrenched disparities in the healthcare system [14]. Implicit bias among providers may also have led to underdiagnosis in children of color [15]. By contrast, our finding of similar prevalences of testing across sociodemographic groups suggests that referral patterns and access to testing were relatively equal within the limits of availability at the time. Differences in test positivity rates are therefore a more valid representation of the unequal disease burden among various sociodemographic strata of the pediatric population, suggesting higher rates among children from Hispanic and lower income households, especially where an adult is an essential worker or has a job that puts them at high risk for COVID-19.

The list of COVID-19 symptoms included in our survey was based on what was known about the disease in children at the time and did not include certain symptoms that emerged as the world’s understanding developed, such as chilblains and rashes [16]. The differing symptom patterns we observed by child age may partially reflect the inability of younger children to communicate certain symptoms, but may also indicate that older and younger children have distinct symptom profiles and that COVID-19 screening should be adapted to account for child age. Because most of our data were collected when testing was by referral only, asymptomatic cases were rarer in our study than reported in the general population; a recent meta-analysis of 18 studies, most of which were of children screened on admission to healthcare facilities, reported a pediatric asymptomatic rate of 46.7% (95% confidence interval: 32.0–62.0%) [17].

While this study provides a unique picture of diagnosis patterns in the early months of the pandemic, it is simultaneously limited by the lack of available testing and by incomplete knowledge about unique pediatric presentations of COVID-19 that characterized the initial phase of the outbreak. Our analysis also relies on participants’ reports of children’s symptoms, testing, and diagnosis rather than medical records. Because we asked participants to report children’s COVID-related information since 1 March, there could be some misclassification error due to inaccurate recall, especially among data collected from those who completed the survey months later, although this represented a small portion of our respondents. Finally, despite having data on large and diverse group of children, we ascertained relatively few cases of COVID-19 and hence had inadequate power to detect statistically significant differences in diagnosis and testing according to many of the sociodemographic metrics that interested us. In particular, we observed few cases among children of women who self-identified as non-Hispanic Black, so were unable to draw inferences based on these categories.

The main strength of this study is that it is nested within the well-characterized NYU CHES cohort, which with few exceptions is reflective of the population of mothers of young children—and by extension, their families—living in NYC. By asking participants to report on all children age ≤18 years living in their households, we were able to expand beyond those children enrolled in NYU CHES (generally 1–2 per family, maximum age 3 years) and describe symptoms and diagnosis patterns across the pediatric age spectrum.

## 5. Conclusions

This snapshot of pediatric COVID-19 cases and symptoms taken in one of the hot spots of the pandemic among a diverse group of participants demonstrates that early in an outbreak, when the availability of objective diagnostic tools such as testing is restricted, subjective case ascertainment methods, such as via symptoms and provider diagnoses, may misrepresent the true prevalence of disease among sociodemographic subgroups. Specifically, we found that children who lived with non-Hispanic White women and women with higher socioeconomic status were more likely to be classified as COVID-19 cases based on reported symptoms or provider diagnosis, while there was a trend toward increased diagnosis of children from less privileged circumstances via testing. Furthermore, pediatricians should be aware that symptom patterns may vary among children of different ages and that these variations should be taken into account when determining diagnostic criteria.

## Figures and Tables

**Table 1:** Prevalence of COVID-19 cases and testing among children age ≤18 years according to sociodemographic factors, NYU CHES, 20 April–31 August 2020.

	N	WHO Suspect Case	Healthcare Diagnosed	Tested Positive ᵃ	Case by Any Method ᵇ	Tested ᵃ	Positive Test Rate ᵃ^,^ᶜ
Total	2694	138 (5.1)	77 (2.9)	12 (0.4)	185 (6.9)	82 (3.0)	14.6%
Child age (years)							
<1	508	21 (4.1)	22 (4.4)	5 (1.0)	34 (6.7)	18 (3.5)	27.8%
1 to 3	1166	65 (5.6)	37 (3.2)	3 (0.3)	87 (7.5)	34 (2.9)	8.8%
4 to 9	581	28 (4.8)	9 (1.6)	1 (0.2)	33 (5.7)	16 (2.8)	6.3%
10 to 18	376	23 (6.1)	7 (1.9)	3 (0.8)	28 (7.5)	14 (3.7)	21.4%
*p*-value		0.51	0.02	0.09	0.55	0.76	0.19
Women’s race/ethnicity							
Hispanic	1565	67 (4.3)	33 (2.1)	10 (0.6)	90 (5.8)	48 (3.1)	20.8%
Non-Hispanic White	726	51 (7.0)	36 (5.0)	1 (0.1)	73 (10.1)	18 (2.5)	5.6%
Non-Hispanic Black	119	4 (3.4)	3 (2.6)	0 (0.0)	5 (4.2)	4 (3.4)	0.0%
Non-Hispanic Asian	200	8 (4.0)	3 (1.5)	1 (0.5)	8 (4.0)	6 (3.0)	16.7%
Other/Multiple	78	8 (10.3)	2 (2.6)	0 (0.0)	9 (4.2)	6 (3.4)	0.0%
*p*-value		0.01	0.004	0.49	0.0003	0.20	0.70
Insurance type							
Public	1622	64 (3.9)	26 (1.6)	6 (0.4)	83 (5.1)	40 (2.5)	15.0%
Private	1034	71 (6.9)	48 (4.7)	4 (0.4)	98 (9.5)	35 (3.4)	11.4%
*p*-value		0.001	<0.0001	1.00	<0.0001	0.16	0.65
Annual household income							
<$30,000	568	16 (2.8)	8 (1.4)	5 (0.9)	24 (4.2)	18 (3.2)	27.8%
$30,000 to $100,000	476	31 (6.5)	19 (4.1)	2 (0.4)	41 (8.6)	21 (4.4)	9.5%
≥$100,000	776	57 (7.3)	37 (4.8)	1 (0.1)	77 (9.9)	18 (2.3)	5.6%
Don’t know	759	30 (4.0)	12 (1.6)	4 (0.5)	38 (5.0)	22 (2.9)	18.2%
*p*-value		0.0005	0.0001	0.23	<0.0001	0.24	0.24
Women’s education							
High school or less	1159	25 (2.2)	23 (2.0)	7 (0.6)	60 (5.2)	33 (2.8)	21.2%
Some college	414	18 (4.3)	10 (2.5)	2 (0.5)	23 (5.6)	14 (3.4)	14.3%
Bachelor’s degree	477	20 (4.2)	13 (2.7)	3 (0.6)	26 (5.5)	17 (3.6)	17.6%
Postgraduate degree	578	55 (9.5)	30 (5.2)	0 (0.0)	73 (12.6)	15 (2.6)	0.0%
*p*-value		<0.0001	0.003	0.24	<0.0001	0.77	0.61
Adult with high-risk job							
Yes	656	34 (5.2)	26 (4.0)	7 (1.1)	49 (7.5)	24 (3.7)	29.2%
No	2038	104 (5.1)	51 (2.5)	5 (0.2)	136 (6.7)	58 (2.8)	8.6%
*p*-value		0.94	0.05	0.01	0.48	0.29	0.045

Unless otherwise specified, data reported as N (row %), ^a^ polymerase chain reaction or antibody testing, ^b^ met WHO criteria, were diagnosed by a healthcare provider, or tested positive, ^c^ (# tested positive)/(# tested).

## Data Availability

The data presented in this study are available upon request from the corresponding author.

## Supplementary Materials

The following are available online at https://www.mdpi.com/article/10.3390/ijerph182211886/s1, Table S1: Characteristics of the first 2000 NYU CHES participants with live deliveries vs. participants in the COVID-19 substudy; Table S2: Prevalence of COVID-19 cases and testing among children age ≤ 18 years, NYU CHES, 20 April–31 August 2020 excluding those who responded after 1 June and who were living outside the NYC metropolitan area; Figure S1: Reported symptoms among children in NYU CHES, 20 April–31 August 2020.
