# Supplementary material for: COVID-19 Symptoms and Diagnoses among a Sociodemographically Diverse Cohort of Children from New York City: Lessons from the First Wave, Spring 2020
Source: Int J Environ Res Public Health. 2021 Nov 12;18(22):11886. doi: 10.3390/ijerph182211886 (PMC8623025; doi:10.3390/ijerph182211886)
Supplement: Supplementary file 1 [file ijerph-18-11886-s001.zip › ijerph-1410331-supplementary.pdf]

ijerph-1410331COVID-19 Symptoms and Diagnoses among a Sociodemographically Diverse Cohort of Children from New York City: Lessons from the First Wave, Spring 2020

Table S1. Characteristics of the first 2000 NYU CHES participants with live deliveries vs. participants in the COVID-19 substudy

	NYU CHES	COVID-19 substudy
	N=2,000	N=1,560
Maternal age	31.8 (5.6)	32.1 (5.6)
Race/ethnicity		
Hispanic	49%	50%
Non-Hispanic White	33%	33%
Non-Hispanic Black	6%	5%
Non-Hispanic Asian	9%	9%
Non-Hispanic Other	1%	1%
Multiple	2%	2%
Marital status		
Married/partnered	88%	88%
Divorced/separated	2%	2%
Single/widowed	10%	9%
Education		
HS or less	32%	33%
Some college	12%	11%
Associate	5%	4%
Bachelor's	23%	24%
Postgraduate	28%	28%
Income		
<$30K	17%	18%
$30 to <$50K	8%	7%
$50 to <$75K	7%	6%
$75 to <100K	5%	5%
$100K or more	38%	40%
Don't know	25%	24%
Employed	65%	65%
Insurance		
Public	52%	50%
Private	48%	50%

NYU CHES: New York University Children's Health and Environment Study

Table S2. Prevalence of COVID-19 cases and testing among children age ≤18 years, NYU CHES, April 20-August 31, 2020 excluding those who responded after June 1 and who were living outside the NYC metropolitan area

	N	WHO suspect case	Healthcare diagnosed	Tested positive[a]	Case by any method[b]	Tested[a]	Positive test rate[a c]
Total	1889	99	53	4	130	37	10.8%
Child age (years)							
<1	351	15 (4.3)	13 (3.8)	2 (0.6)	21 (6.0)	11 (3.1)	18.2%
1 to 3	774	46 (5.9)	26 (3.4)	0 (0.0)	61 (7.9)	11 (1.4)	0.0%
4 to 9	443	22 (5.0)	7 (1.6)	0 (0.0)	26 (5.9)	8 (1.8)	0.0%
10 to 18	275	16 (5.8)	5 (1.8)	2 (0.7)	20 (7.3)	7 (2.6)	28.6%
p-value		0.66	0.14	0.047	0.50	0.25	0.25
Women's race/ethnicity							
Hispanic	1174	53 (4.5)	21 (1.8)	3 (0.3)	67 (5.7)	25 (2.1)	12.0%
Non-Hispanic White	449	33 (7.4)	24 (5.4)	0 (0.0)	48 (10.7)	5 (1.1)	0.0%
Non-Hispanic Black	83	2 (2.4)	3 (3.7)	0 (0.0)	3 (3.6)	2 (2.4)	0.0%
Non-Hispanic Asian	135	7 (5.2)	3 (2.2)	1 (0.7)	7 (5.2)	5 (3.7)	20.0%
Other/Multiple	45	4 (8.9)	2 (4.7)	0 (0.0)	5 (11.1)	0 (0.0)	0.0%
p-value		0.099	0.003	0.54	0.003	0.29	0.89
Insurance type							
Public	1191	52 (4.4)	15 (1.3)	2 (0.2)	61 (5.1)	23 (1.9)	8.7%
Private	678	47 (6.9)	38 (5.7)	2 (0.3)	69 (10.2)	13 (1.9)	15.4%
p-value		0.02	<0.0001	0.57	<0.0001	0.98	0.59
Annual household income							
<$30,000	444	14 (3.2)	5 (1.1)	2 (0.5)	19 (4.3)	12 (2.7)	16.7%
$30,000 to $100,000	317	22 (6.9)	12 (3.8)	0 (0.0)	28 (8.8)	7 (2.2)	0.0%
≥$100,000	487	38 (7.8)	29 (6.0)	1 (0.2)	54 (11.1)	6 (1.2)	16.7%
Don't know	569	21 (3.7)	6 (1.1)	1 (0.2)	24 (4.2)	12 (2.1)	8.3%
p-value		0.002	<0.0001	0.61	<0.0001	0.45	0.73
Women's education							
High school or less	892	31 (3.5)	13 (1.5)	3 (0.3)	39 (4.4)	19 (2.1)	15.8%
Some college	272	16 (5.9)	8 (3.0)	0 (0.0)	20 (7.4)	6 (2.2)	0.0%
Bachelor's degree	323	13 (4.0)	7 (2.2)	1 (0.3)	17 (5.3)	6 (1.9)	16.7%
Postgraduate degree	361	37 (10.3)	24 (6.7)	0 (0.0)	51 (14.1)	6 (1.7)	0.0%
p-value		<0.0001	<0.0001	0.55	<0.0001	0.94	0.61
Adult with high-risk job							
Yes	401	25 (6.2)	16 (4.1)	2 (0.5)	33 (8.2)	6 (1.5)	33.3%
No	1488	74 (5.0)	37 (2.5)	2 (0.1)	97 (6.5)	31 (2.1)	6.5%
p-value		0.31	0.10	0.16	0.23	0.45	0.11

Unless otherwise specified, data reported as N (row %)

[a] polymerase chain reaction or antibody testing

[b] met WHO criteria, were diagnosed by a healthcare provider, or tested positive

[c] (# tested positive)/(# tested)

a. Children diagnosed via WHO suspect case criteria (n=138).

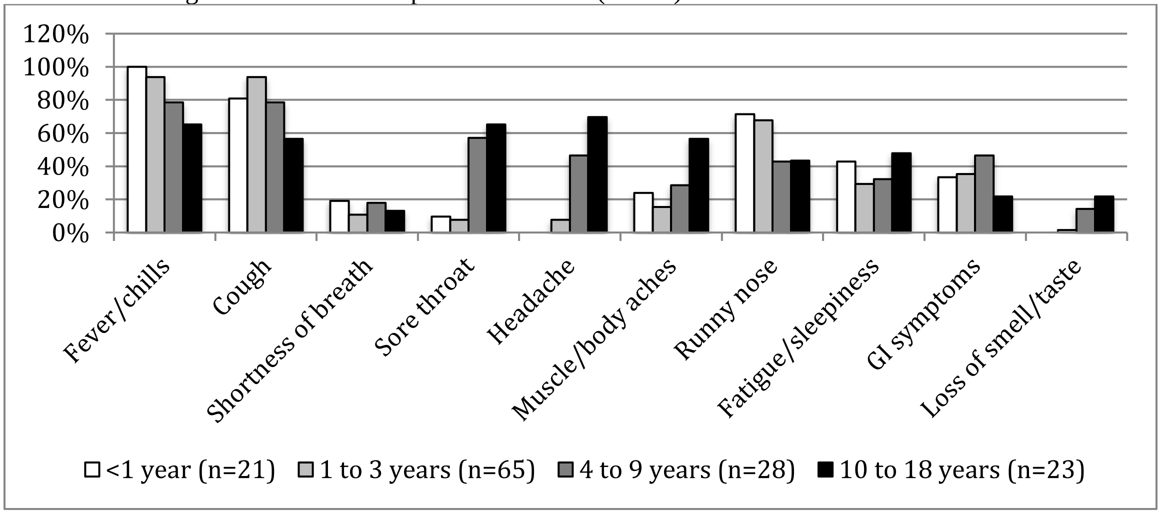

b. Children diagnosed by a healthcare provider (n=77).

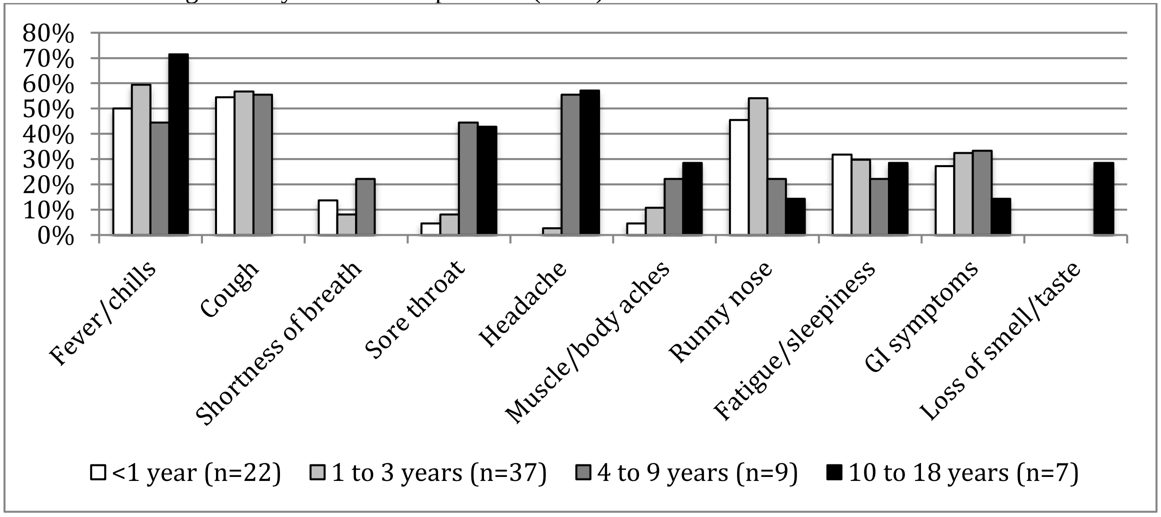

c. Children who tested positive (n=12).

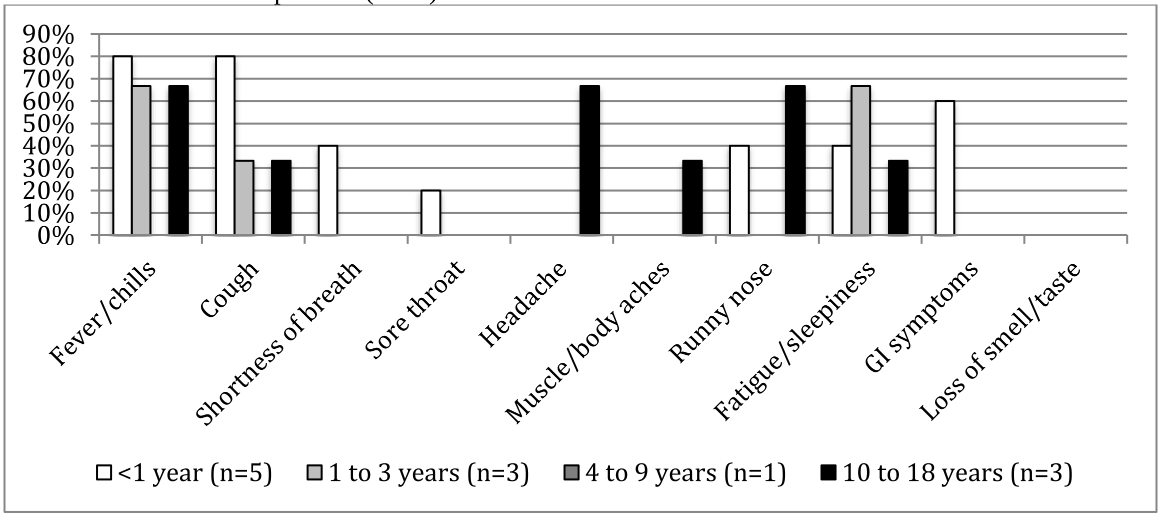

d. Children without a diagnosis (n=2509).

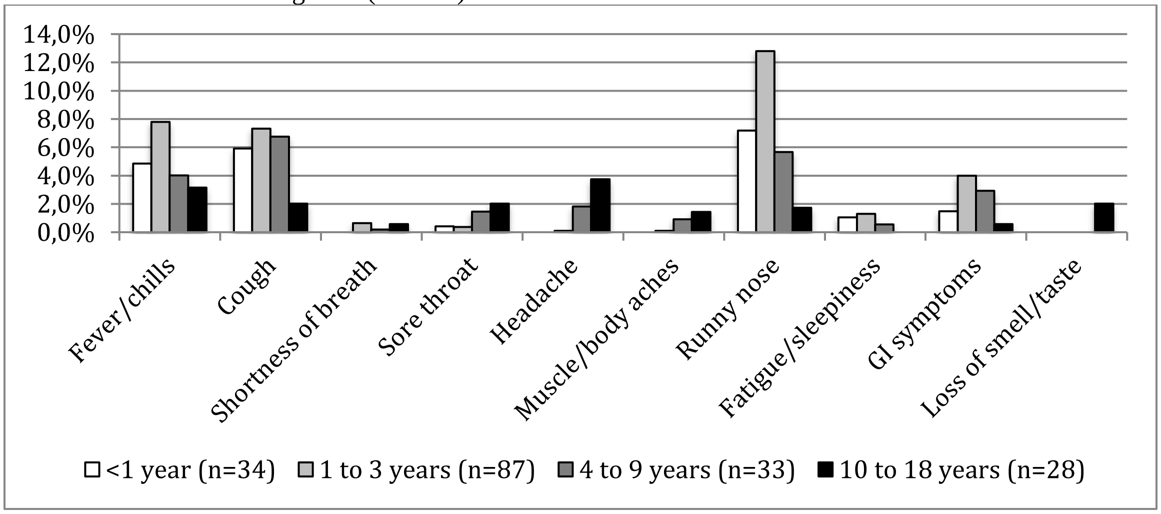

e. Children with and without diagnoses, all age groups combined.

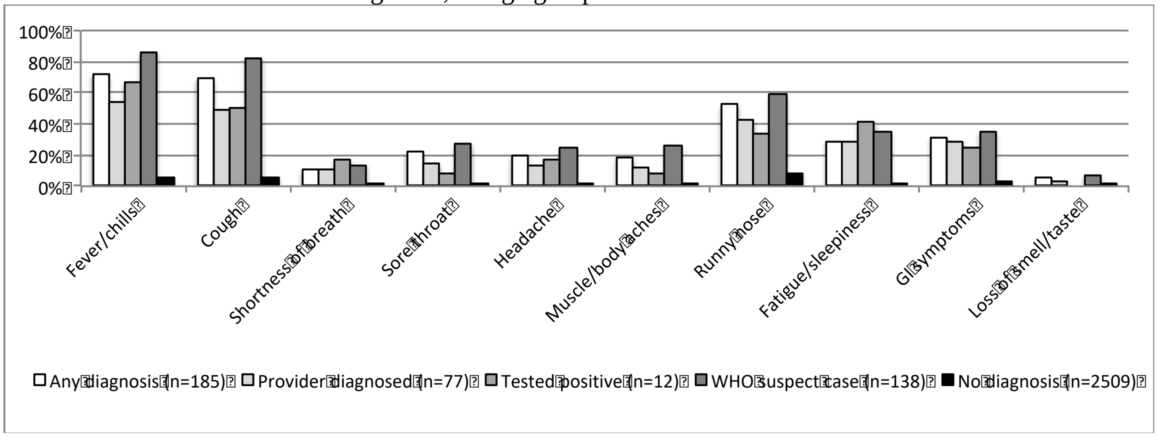

Figure S1. Reported symptoms among children in NYU CHES, April 20-August 31, 2020.
